# Guidelines of the Brazilian Dermatology Society for diagnosis, treatment and follow up of primary cutaneous melanoma - Part I*

**DOI:** 10.1590/abd1806-4841.20154707

**Published:** 2015

**Authors:** Luiz Guilherme Martins Castro, Maria Cristina Messina, Walter Loureiro, Ricardo Silvestre Macarenco, João Pedreira Duprat Neto, Thais Helena Bello Di Giacomo, Flávia Vasques Bittencourt, Renato Marchiori Bakos, Sérgio Schrader Serpa, Hamilton Ometto Stolf, Gabriel Gontijo

**Affiliations:** 1 Hospital Israelita Albert Einstein - São Paulo (SP) Brazil; 2 Hospital Alemão Oswaldo Cruz - São Paulo (SP) Brazil; 3 Oncoderma - São Paulo (SP) Brazil; 4 Hospital Ipiranga - São Paulo (SP) Brazil; 5 Universidade Estadual do Pará (UEPA) - Belém (SP) Brazil; 6 Instituto do Câncer do Estado de São Paulo (ICESP) - São Paulo (SP) Brazil; 7 Departamento de Câncer de Pele A. C. Camargo Cancer Center - São Paulo (SP) Brazil; 8 Universidade Federal de Minas Gerais (UFMG) - Belo Horizonte (MG) Brazil; 9 Universidade Federal do Rio Grande do Sul (UFRGS) - Hospital de Clínicas de Porto Alegre, Porto Alegre (RS), Brazil; 10 Universidade Estácio de Sá (UNESA) - Rio de Janeiro (RJ) Brazil; 11 Universidade Estadual Paulista "Júlio de Mesquita Filho" (UNESP) - Botucatu (SP), Brazil

**Keywords:** Dermoscopy, Diagnostic imaging, Diagnostic techniques and procedures, Guideline, Histology, Melanoma, Practice guideline, Sentinel lymph node biopsy, Therapeutics

## Abstract

The last Brazilian guidelines on melanoma were published in 2002. Development in diagnosis and treatment made updating necessary. The coordinators elaborated ten clinical questions, based on PICO system. A Medline search, according to specific MeSH terms for each of the 10 questions was performed and articles selected were classified from A to D according to level of scientific evidence. Based on the results, recommendations were defined and classified according to scientific strength. The present Guidelines were divided in two parts for editorial and publication reasons. In the first part, the following clinical questions were answered: 1) The use of dermoscopy for diagnosis of primary cutaneous melanoma brings benefits for patients when compared with clinical examination? 2) Does dermoscopy favor diagnosis of nail apparatus melanoma? 3) Is there a prognostic difference when incisional or excisional biopsies are used? 4) Does revision by a pathologist trained in melanoma contribute to diagnosis and treatment of primary cutaneous melanoma? What margins should be used to treat lentigo maligna melanoma and melanoma in situ?

## INTRODUCTION

Cutaneous melanoma (CM) is one of the most potentially dangerous forms of skin cancers, accounting for approximately 90% of deaths. The dermatologist is in the forefront of diagnosis and treatment of CM. It is his duty to keep updated on best practices in diagnosis, treatment and disease monitoring to be able to diagnose, treat and council patients in the best way. The last Brazilian guidelines on CM were published in 2002.^1^ Over 10 years have passed and important advances in the area occurred during this period, with greater relevance to diagnostic techniques. Although some concepts have not changed, it is necessary to update the standards of practice on this important health problem.

These guidelines are intended for diagnostic and therapeutic approach and follow-up of patients with suspected or confirmed diagnoses of primary CM (PCM) with no clinical nor histological evidence of metastatic disease (stages 0, I and II). They do not include ocular nor mucosal melanoma.

## OBJECTIVE

To introduce the most advanced evidences in diagnosis, therapeutic management and monitoring of PCM clinical stages 0, I and II patients, describing diagnostic peculiarities that allow identification of these tumors in early stages, as well as measures most widely accepted for treatment and follow-up in the context of Brazilian dermatology.

## METHOD - DESCRIPTION OF EVIDENCE COLLECTION

The coordinators have defined 10 questions that reflect issues of clinical relevance on the subject. The questions were structured according to the acronym *PICO* (patient or population, intervention, comparison or control and outcome), according to regulations of the National Health Agency, the Brazilian Medical Association and Federal Council of Medicine, described in "The process of development, validation and implementation of clinical guidelines in the private healthcare system in Brazil".^2^ To answer these questions, a literature review of scientific articles was conducted in MEDLINE database. Search for evidence was limited primarily to articles published between 01/01/2009 and 06/30/2014 and keywords (MeSH terms) present in the title and/or summary were used, grouped into specific syntaxes for each of the 10 questions alone, as described in Chart 1.

**Chart 1:** Terms of the descriptors used to research each question and number of selected articles

**Question**	**Terms**	**Number of articles**
1	(dermoscopy OR dermatoscopy) AND melanoma AND diagnosis	339
2	(dermoscopy OR dermatoscopy) AND melanoma AND (nail OR subungual)	21
3	(excisional OR incisional) AND melanoma AND biopsy AND prognosis	13
4	pathology review AND melanoma	9
5	(therapy OR treatment) AND (lentigo maligna OR melanoma in situ)	903

After reading the abstracts, articles containing information relevant to the subject were selected. When appropriate, references present in these papers were selected, without limit for publication period, and were analyzed using the same methodology as that for the primarily selected studies.

Articles that presented the expected contribution were analyzed regarding the level of evidence (Table 2). Recommendations were written in response to the questions elaborated. Recommendations were also graduated according to level of evidence (Chart 2).

**Chart 2:** Grade of recommendation and level of evidence:

A: Experimental or observational studies of higher consistency.
B: Experimental or observational studies of lower consistency.
C: Case reports / uncontrolled studies.
D: Opinion without critical evaluation, based on consensus, physiological studies or animal models.

## RESULTS AND DISCUSSION

Discussion and recommendations for the first 5 questions appear below. The other 5 questions will be presented in a new publication.

1) Is the use of dermoscopy for diagnosis of PCM beneficial for patients with suspicious lesions when compared to clinical examination?

For many years dermatologists could only rely on clinical diagnosis when facing a suspicious melanoma lesion. Implications of such diagnosis justified extreme measures such as removal of large amounts of suspicious lesions, which subsequently revealed to be benign (unnecessary resections).

A major step in improving accuracy of diagnosis of primary cutaneous melanoma (PCM) occurred with the introduction of "Melanoma ABCD" rule, acronym introduced in 1985, using the initials of four clinical features of skin lesions that may be indicative of malignancy (Asymmetry, irregular Borders, varied Colors and Diameter >6 mm).^3^ Although increasing diagnosis sensitivity and specificity, the number of nevus excised for diagnosing a melanoma was still high. A few years later the letter "E" was included in the acronym to represent "*E*volution" or changes in the appearance of a suspicious lesion.^4,5^

Dermoscopy (DMCP) enables the examination of skin lesions, melanocytic or not, and the observation of structures and colors of the epidermis, dermo-epidermal junction and superficial dermis, not visible to the naked eye. These findings correlate with histological features and are used to determine whether the lesion is malignant or benign and if surgical removal is indicated. When integrated with information obtained from the clinical history and macroscopic examination, DMCP allows obtaining such a large amount of information, that it has gradually became an irreplaceable instrument for the dermatologist, such as the stethoscope is for the clinician.

Naked eye diagnostic sensitivity for PCM (percentage of correctly diagnosed melanomas) is around 71% whereas with the use of dermatoscope is 90%. A meta-analysis of 22 studies showed that diagnostic accuracy was higher than naked eye examination when experts used DMCP (sensitivity 89% and specificity 79%) (level of evidence A).^6^ There was no decrease in specificity, suggesting that the DMCP increases diagnostic accuracy without increasing the number of undiagnosed melanomas (level of evidence A).^7,8^

To achieve these higher efficiency levels it is necessary to use an algorithm for analyzing suspicious skin lesions. The 4 best known are: Menzies' scoring method; pattern analysis; 7-points check list; and ABCD of DMCP, which - it's important to note - is different from the clinical ABCD.^9^ At a consensus DMCP experts meeting, standard analysis algorithm reached the highest specificity, sensitivity and accuracy for melanoma detection when compared with the other 3 algorithms (level of evidence B).^10^ Any of these algorithms presents higher effectiveness than examination with naked eye (level of evidence A).^7,11-13^ In summary, when using DMCP to evaluate any suspicious lesion, compared with evaluation by the naked eye, the probability of classifying an actual malignant lesion as malignant is higher, as well as classifying as benign a truly benign lesion. At this point it is important to remember that even with the use of DMCP accuracy of diagnosis melanoma does not reach 100%. This is due to the lack of dermoscopic features suggestive of the diagnosis in very early lesions or in some cases of uncharacteristic melanomas (featureless).^6,14^

In addition to increased PCM diagnostic accuracy, use of DMCP presents 2 more clear benefits: reduction of unnecessary resections and increased diagnosis of non-melanoma skin cancer (level of evidence A).^15-18^ A randomized study showed 42% decrease in the number of unnecessary biopsies.^16^ This finding is similar to a retrospective study of excisions of pigmented lesions performed before and after training in DMCP. The rate of benign/malignant lesions decreased from 18.0: 1 to 4.3:1 (level of evidence A).^16^

Importantly, the effectiveness DMCP use depends on formal training and experience. It was demonstrated that dermatologists with formal training and at least 3-years experience in DMCP reach higher CM detection rates than non-trained dermatologists (level of evidence B).^19^ This learning process is time consuming and in the early stages of the learning curve worsening of accuracy can occur.^20^ Dermatologists, physicians from other specialties, paramedical staff and even medical students have consistently improved diagnosis accuracy after being trained in DMCP use (level of evidence A). A considerable number of dermatologists believe that the time required for training in DMCP is excessive.^21^ At the same time, some studies show a low level of satisfaction among chief-residents of dermatology in relation to training in DMCP. Many want more intense instruction in the area. There are also complaints about the limited availability of courses (level of evidence C).^22^

**Recommendations:**
- DMCP increases diagnostic accuracy of PCM compared to clinical examination. It should be used routinely in the evaluation of melanocytic lesions (grade of recommendation A);
- The use of DMCP for this purpose requires specific training in order to achieve the desired accuracy (grade of recommendation A).
- In addition to being useful in diagnosing PCM, regular use of DMCP also has the advantage of reducing the number of unnecessary excisions (grade of recommendation A);
- In addition to being useful in diagnosing PCM and reducing the number of unnecessary excisions, regular use of DMCP leads to an increase in diagnosis of non-melanoma skin cancer (grade of recommendation A).

2) Does dermoscopy favor diagnosis of primary cutaneous melanoma of the nail apparatus (NA)?

NA-PCM represents 0.7% to 3.5% of all melanomas.^23,24^ Although deemed little frequent when considering that the palm is equivalent to 1% of the body surface area and the total area of the 20 nails is much smaller than the area of the palm, it becomes clear the incidence of NA-PCM is proportionally greater than in the rest of the body.^25^ In Caucasians NA-PCM responds from 1,4 to 2,0% of all melanomas.^26,27^ In Afro-american population it is more frequente, reaching 20% of all melanoma. In American indians frequency may be even higher (31%).^28-30^ In no ethnic group the frequency of NA-PCM is as high as in japanese population. In this population, acral melanoma (which includes all NA-PCM) responds for 50 to 77% of all melanomas.^31,32^.

Early diagnosis of NA-PCM improves prognosis. However, biopsies, essential for correct diagnosis and staging, carry the possibility of permanent dystrophies of the NA. The inherent complexity of surgical procedures in the NA delays great part of melanoma diagnosis in this location, closing the opportunity window for curative treatment. Lesions arousing much suspicion, such as large lesions or lesions causing nail dystrophy, are usually biopsied. However, these are usually associated with more advanced stages and worse prognosis.

Clinically, subungual melanoma (SUM) at initial stages is often characterized as striated melanonychia (SM), defined as dark bands growing in the longitudinally on the nail. Since it is not a pathognomonic NAPCM clinical finding, melanoma associated SM should be differentiated from those of non-malignant etiology. Early differential diagnosis of SM still remains a challenge for dermatologists, even for nails experts (level of evidence B).^33^ Over the years, signs and methods to assist the physician in early diagnosis of NA melanoma of have been sought. Among them are:

a) Clinical examination:

Hutchinson's signal (HS), periungual pigmentation with or without SM, is historically considered as the most important clinical sign to differentiate SUM from benign melanocytic nevi.^34,35^ However, it is not a pathognomonic sign since it is not present in amelanotic melanoma and can be seen in benign conditions such as melanocyte activation and even in benign melanocytic nevi (level of evidence A).^36^ HS should be differentiated from pseudo-HS (visualization of pigmentation in nail bed or matrix due to peri-ungual tissue transparency) that may be present in Bowen's disease and in benign conditions that cause subungual hyperpigmentation.^37,38^ Topical or systemic drugs can also cause SM, which may or may not be associated with the peri-ungual pigmentation.^39-42^

Clinical examination with naked eye was used for decades as the sole diagnostic method. To increase accuracy, ABCDEF rule for SUM was created, like the ABCD rule for CM.^43,44^ Each letter refers to a characteristic associated with increased risk for SUM, as it is shown in Chart 3.

**Chart 3:** Meaning of the mnemonic method "ABCDEF" of melanoma of the nail apparatus

Letter	Meaning	Detailing
Age	Most affected age and ethnicities	From 20 to 90 years, with peak of incidence between the 5th and 7th decades;
		African-Americans, Asians and American Indians.
Band	Band or stripe	Striation width equal to or greater than 3mm
Change	Change	Rapid growth in the size of the band;
		Absence of recovery (healing) of nail dystrophy despite treatment.
Digit	Finger involved	Order of incidence: pollex > hallux > index; single finger > several fingers; dominant hand.
Extension	Extension	Pigment extension to proximal, lateral or hyponychium nail fold (Hutchinson's sign).
Family	Family history	Family/ personnel history of melanoma or dysplastic nevus syndrome.

b) Nail plate dermoscopy:

DMCP plays a key role in PCM early diagnosis, however, this role is less impacting on the evaluation of SM and SUM. It's important to remember that DMCP helps to differentiate melanin and blood (although it cannot differentiate between bleeding caused by tumor). There is no evidence of the superiority of polarized in relation to non-polarized light in the examination of SM.^42^

c) Intraoperative dermoscopy:

The nail plate is translucent and allows dermoscopic visualization of the pigment present in the nail bed, but visual characteristics are altered, making it difficult to reach a correct interpretation. If the nail plate is surgically removed, it is possible to perform direct DMCP on the nail bed using a polarized light dermatoscope.

Studies correlating nail bed DMCP with histological evaluation of SM allowed identifying patterns with high diagnostic accuracy (level of evidence A).^43^ It is important to note that, intraoperative DMCP does not replace histologic examination, which remains the gold standard for diagnosis of SUM and provides crucial data for evaluation of surgical margins, therapy and prognosis (level of evidence B).

Tangential excision, also known as "nail matrix shave biopsy" allows the removal of lesions larger than 3 mm with minimal risk of scarring the NA, as well as enabling an assessment of safety margin (level of evidence B).^25,42-44^

Recognizing and biopsying SUM in early stages is key to achieving cure rates similar to those of *in situ* PCM.

**Recommendations:**
- ABCDEF rule provides a methodology for clinical evaluation of SM and increases clinical diagnosis sensitivity of SUM (grade of recommendation A).
- nail plate DMCP increases accuracy for indicating SM biopsies (grade of recommendation A).
- Intraoperative DMCP has high level of accuracy in the correlation between visualized and histological pattern (grade of recommendation A).
- Histologic examination still remains the gold standard for definitive diagnosis of NA melanoma (grade of recommendation A).

3) Is there prognostic difference when performing incisional or excisional biopsy of suspected melanoma lesions?

Excisional biopsy (EB) with 1 to 3 mm margins is recommended as first choice therapy for pigmented lesion suspected of melanoma. EB enables better histological evaluation, which includes: tumor thickness (Breslow depth), MIand presence or absence of ulceration. These 3 items are essential to define staging, prognosis and treatment.^45^ There are situations where EB is difficult to perform, such as, but not only extensive pigmented lesions on the face, mucous membranes or acral lesions or extensive lesions with low suspicion of melanoma. In such cases, incisional biopsy (IB) would be less invasive than EB.^46^

Although IB is technically simpler to perform and has lower cost, a point that should be taken into consideration since healthcare resources are scarce in many countries, it allows only a partial histological evaluation of the lesion.^46^ The concern that mechanical manipulation (such as punch biopsy or needle biopsy) could implant neoplastic cells and increase the risk of metastases was great for many years and IB was contraindicated at that time. This potential risk has been discussed not only for melanoma, but also for various types of tumors.47 Presently it is believed this risk has been overestimated and there are considerations that tumor cells molecular characteristics, such as adhesion molecules, matrix metalloproteinase and vascular growth factors, may influence the ability to migrate and to induce vascularization, which would be more important in disseminating tumor cells than just mechanical manipulation.^48^

To assess whether IB resulted in worse prognosis compared to EB, several retrospective and prospective studies were conducted.^49-57^ Most of them, especially those with larger numbers of patients, showed that the type of biopsy does not play a role in the evolution of melanoma patients (level of evidence A).

IB can be performed by shave, punch or incisional techniques. A study comparing histological evaluation of EB, shave biopsy, punch biopsy and IB, showed that treatment recommendations changed in 6% of the cases: (2% after EB, 5% after shave biopsy, 18% after punch biopsy and 18% after IB). Thus, it was found that EB allows better histological evaluation and should be preferred whenever possible (level of evidence A).^58^

A prospective study designed to evaluate the impact on staging, area of margins expansion, sentinel node positivity, tumor recurrence and survival evaluated 709 patients who underwent punch biopsy (23%), shave biopsy (34%) and EB (43%). There was no significant difference in accuracy of sentinel lymph node results, tumor recurrence nor in patient survival. The study concluded that biopsy type did not impact SLNB accuracy or results, tumor recurrence, or prognosis. Punch and shave biopsies, when used appropriately, should not be discouraged for the diagnosis of melanoma (level of evidence A).^59^

IB may be used for lesions suspected of melanoma located in areas where EB is difficult to perform, such as - but not limited to - large lesions, lesions located on the face, mucous membranes and acral regions or lesions with low probability of melanoma (level of evidence B).^46^

When deciding on IB, it must be performed in locations that allow histological evaluation of the greater depth of the lesion (clinically darker areas and/or papular). DMCP can be a useful tool for selecting the best place for biopsy, but there are still no studies with large number of patients that support this recommendation (level of evidence D).^60^

**Recommendations:**
- EB should be the preferred technique whenever possible, because it allows better tumor histological evaluation, which directly impacts in the conduct and prognosis (grade of recommendation A).
- IB does not affect prognosis of melanoma patients and can be used when necessary (grade of recommendation A).
- IB is suggested in the following cases, but is not limited to (grade of recommendation B):
- Extensive pigmented lesions with low suspicion of melanoma;
- Suspected extensive lentigo maligna on the face;
- Suspicious pigmented lesions, extensive or in an acral region;
- Mucosal lesions.
- The major axis of the biopsy should be oriented so as to facilitate possible margins expansion. In general, when performed in the members it means along the major axis (grade of recommendation C).

4) Does the review of histological findings by a pathologist trained in melanoma contribute to the diagnosis and treatment of PCM?

The gold-standard for PCM diagnosis is histopathology. Roughly, there exist about 20 histological items to be evaluated in each case before a final diagnosis is rendered. This is a challenging task, which can be influenced by interpretation biases^61^. Experience and interest of general pathologists on this issue are heterogeneous, as is the frequency such a neoplasm is found in diagnostic routine in general pathology laboratories.

In addition to correct diagnosis, it is essential that histologic reports on melanoma address microstaging (MST) items, which will be essential for determining treatment and prognosis^62^. Since appropriate local treatment and the decision of performing sentinel lymphnode biopsy rely on MST items, histologic report must include at least the following data^8,62-68^:

1. Margin involvement and margin distances from the neoplasm;
2. Presence or not of dermal invasion;
3. In invasive neoplasms:

- Tumor thickness (Breslow depth);
- Mitotic index (MI);
- Ulceration: present or not;
- Microsatellitosis /satellitosis.

Studies have shown that information on MI, ulceration, microsatellitosis and tumor thickness are not originally included in melanoma histologic reports in 47-50%, 13-72%, 50%, and 29% of cases, respectively.^69-71^ In order to reduce such frequencies, some of the most influential pathology societies worldwide created a consensus protocol on mandatory and wishful items to be included in melanoma histologic reports.^66^

Correct histological criteria to determine dermal invasion, ulceration, and microsatellitosis, as well as proper methodology for measuring Breslow thickness and mitotic counting are of equal importance.^66-66^

Physicians reading melanoma histologic reports cannot directly check if proper methodology for each item was used correctly in a given case. The only practical way to achieve this goal is to request a second opinion to a pathologist with expertise in melanoma or to send the slides to a melanoma referral hospital for histopathological review.

Indeed, a recent study has shown that review of thin and in situ melanomas by an expert pathologist has led to changes in diagnosis and microstaging with subsequent modification of original proposed local treatment and SLNB in 12% and 16%, respectively. (Level of evidence B).^69^ Similarly, a retrospective large series showed that margin status was modified in 11% of cases after expert review.^72^

It has been shown that reporting of Breslow thickness, MI, and ulceration has good inter-observer reproducibility among pathologists.^73^ ^-7)^6 On the other hand, microsatellitosis remains a microstaging item of low reproducibility.75 This data seems very important because the detection of microsatellitosis in primary melanoma resection (or biopsy) specimen modifies pathologic staging from pN0 (or pNx) to pN2c.^8,68,69^

With regard to differential diagnoses of melanocytic nevus versus melanoma, studies report that reproducibility ranges from 73% to 97.3% between general and expert pathologists.^72,75-78^ It has also been known for a long time that ambiguous melanocytic lesions can lead to diagnostic disagreement even among experts.^79^ Examples of ambiguous lesions include atypical nevus with severe cytological and architectural atypia, atypical Spitz tumors, etc. Advances in genetics knowledge have brought profound changes in the understanding of these ambiguous lesions. As a result, the polarization "melanocytic nevus (benign) versus melanoma (malignant)" has progressively given way to the recognition of a range of intermediate lesions categorized as low-grade malignant or of uncertain biological behavior. Although such lesions do not constitute a clinicopathological entity, they form a spectrum with intermediate morphological and genetic aberrations lying between classical nevi and melanomas. As a group, these lesions have the potential to evolve with microscopic regional lymphnode metastases, but they rarely lead patients to death 80. Examples of such lesions include atypical Spitz tumors, atypical blue nevi, and melanocytoma (also known as epithelioid blue nevus. which presents some overlap with animal- type melanoma, according to some authors)^80-82^.

Great effort of the scientific community has been made to stratify this intermediary group of lesions by fluorescent in situ hybridization (FISH) so that lesions with higher probability of aggressive behavior may be detected and managed accordingly, particularly atypical Spitz tumors (level of evidence B)^83^. Immunohistochemistry, FISH, and other molecular pathology techniques have been used in order to reduce subjectivity of morphological examination of ambiguous cases (level of evidence B)^84-87^.

**Recommendations:**
- Definite diagnosis of melanoma is based on histopathology of excision or biopsy specimens (level of recommendation A);
- Melanoma histologic report must include histological items of MST (level of recommendation A).
- Histological review by an expert pathologist/melanoma referral center should be sought whenever possible before definitive treatment (level of recommendation B).
- Immunohistochemistry and molecular studies can be used to aid in the diagnosis of selected cases of ambiguous melanocytic lesions (level of recommendation B).

5) What margin should lentigo maligna melanoma and melanoma ***in situ*** be excised with?

The primary treatment for PCM is surgical resection.^88,89^ Historically, through the 20th century, resection with circumferential margin of 3-5 mm was indicated for PCM of any thickness, followed by grafting. Rationale was based on the occurrence of "centrifugal lymphatic spread", seen in histological preparations of excised melanomas.^90^

Current recommendation is that smaller margins are used, reducing surgery morbidity. This is based on prospective studies comparing more conservative with more radical surgeries, on randomized studies and on international consensus conferences.^91-103^ These studies demonstrated that histological findings will determine the appropriate surgical margins, and the Breslow thickness is the most important data (level of evidence A).

It is important to remember that for both *in situ* and invasive melanomas, histological examination with paraffin embedded sections is the gold standard for evaluation of surgical margins and that margins expansion surgery should be performed preferably between 4 to 6 weeks from the initial biopsy (level of evidence C).^100^

Margins recommended for PCM surgical excision appear below (Table 1).

**Table 1:** Surgical Margins for the treatment of primary cutaneous melanoma

Breslow thickness (mm)	Surgical margin (cm)	Level of evidence
In situ	0.5#	A
Up to 1.00	1.0	A
From 1.01 to 2. 00	1.0 to 2.0*	B
More than 2.00	2.0	A

t-test

* The surgical margins can be modified to contemplate anatomical, functional or aesthetic needs. Experts agree that margins between 1cm and 2 cm are acceptable in areas where margins of 2 cm would cause significant aesthetic, functional or anatomical losses. The patient should be informed and agree with the doctor about the best option.

There is no consensus regarding deep margins resection as it occurs for lateral margins. Historically, the recommendation is that invasive PCM is resected until the muscle fascia. There is, however, no evidence confirming that such action is necessary in all cases. A group of experts recommends that, whenever possible, resection is made to the fascia or to the deep subcutaneous tissue, depending on tumor location (level of evidence B).

Although surgical resection is the standard treatment for *in situ* melanoma (isM) and for LM, scientific evidences regarding the surgical margins to be used are not as clear as for invasive melanomas. This is because it is known that large isM, LM and lentigo maligna melanoma (LMM) often present subclinical atypical junctional hyperplasia in peripheral and peri-adnexal areas of the lesion, which can extend for several millimeters beyond the visible margins.^45,101-103^

Several authors agree that 5 mm margins may often be insufficient. Many studies have shown that up to 20% of LM require margins larger than 5 mm, while 12% of LMM with Breslow thickness up to 1.0 mm require margins with more than 10 mm (level of evidence B).^104^ According to Kunishige et al, 9 mm margins would be sufficient in 99% of cases (level of evidence B).^105^

It is also known that recurrent LM and LMM tend to require wider margins (level of evidence B). The lack of prospective studies suggests that, in the cases of large isM, LM and LMM, the determination of the margins is defined by consensus of expert groups or by retrospective studies (level of evidence B).

The usefulness of the various methods of micrographic control of margins is very discussed.^45,101,103^ The most disseminated is Mohs' method, but several other methods of margins micrographic control appear in the literature, with different nomenclatures such as 3D histology method, Tubingen pie chart, staged surgical excision, etc. Micrographic surgery is a very useful method to treat lesions in areas where healthy tissue preservation is important, such as the face, as well as to allow greater surgical margins control. It also diminishes the risk of recurrence and may reduce the final size of surgical wounds (level of evidence C).^45,101,104,106^

At this point it is important to remember that histological examination by conventional frozen sections are not suitable for determining surgical margins of melanocytic lesions (level of evidence A). The ideal is to examine paraffin sections. Advances in IHC techniques towards melanocytes identification will allow frozes sections to achieve higher reliability rates.^107-111^

Some authors recommend the use of Wood's light to assist in assessing the real extent of the tumor (level of evidence D).^112^

Use of alternative non-surgical therapeutic methods for treating LM or *is*M, (imiquimod and radiation) are justified in cases where surgery can cause great aesthetic/functional damage or in patients unable to undergo surgery.^45,104^ Literature presents reports of complete response of up to 88% of cases, but it is important to consider that it is not possible to obtain a complete histological analysis of the lesion, thus eventual invasive melanoma foci would not be identified. Histological examination after treatment showed persistence of disease in up to 25% of treated patients and there were cases of LM progression to invasive melanoma. Moreover, diversity of therapeutic regimens and short monitoring period limit the comparative studies (level of evidence C).^101^

Cryosurgery with liquid nitrogen has not been adequately studied, but it's one more option. There are no comparative long-term studies on the subject (level of evidence D).^101^

- Recommendation
- For patients with isM, surgical margins of 0.5 cm are sufficient (grade of recommendation B).
- Treatment margins for large isM / LM are at least 0.5 cm, and it may be necessary to increase them (grade of recommendation A).
- The use of micrographic surgery techniques for large isM, LM and LMM can assist in more accurate determination of surgical margins, but paraffin sections should be used and, when available, IHC techniques (grade of recommendation B).
- Use of imiquimod to treat LM or isM, although off-label, is justified in cases where surgery can cause great aesthetic/functional impairment or in patients unable to undergo surgery. Because data for these situations are limited, the surgeon must make the decision with the patient (grade of recommendation C).

At the end of this first part of the Brazilian guidelines on melanoma, it is extremely important to point out that they are not intended to stifle medical practice, but to make it more homogeneous, reducing uncertainty/disagreement on good practice standards. Stablishing standards, besides reducing the differences in patient care, can also make possible to provide options based on evidence, allowing the physician to make decisions about treatment or diagnostic methods, thus reducing the strain on patients, doctors and on the healthcare system.

These guidelines reflect the best scientific information published about the subject to the date of its preparation. Nevertheless, we must be careful when interpreting these data, since the outcome of future studies can lead to changes in recommendations. In some cases, it may be necessary not to follow these guidelines, always keeping in mind patients' well-being , as well as other special circumstances.
